# Supplementary material for: Non-obstructive intracellular nanolasers
Source: Nat Commun. 2018 Nov 16;9:4817. doi: 10.1038/s41467-018-07248-0 (PMC6240115; doi:10.1038/s41467-018-07248-0)
Supplement: Supplementary file 1 — Supplementary Information [file 41467_2018_7248_MOESM1_ESM.pdf]

Non-obstructive intracellular nanolasers

Supplementary Information

Alasdair H. Fikouras, Marcel Schubert, Markus Karl, Jothi D. Kumar, Simon J. Powis, Andrea di Falco*, Malte C. Gather*

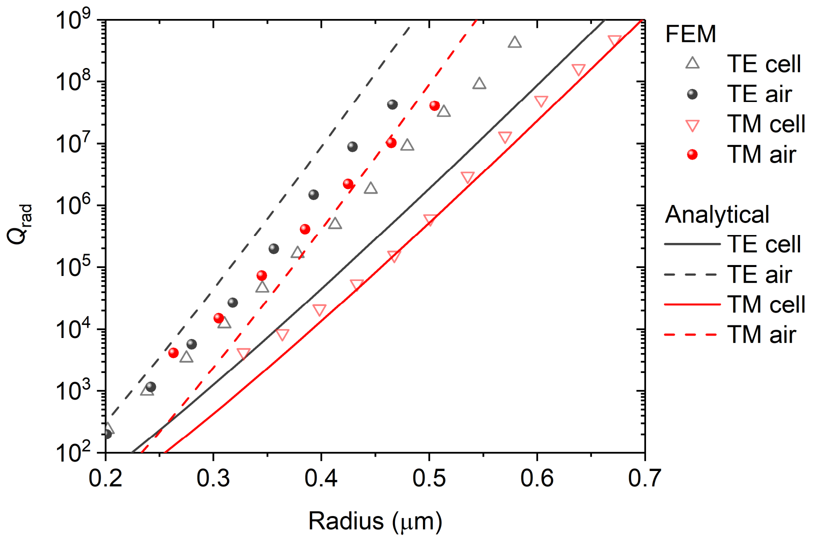

Supplementary Fig. 1. Radiative *Q* factor of lowest radial order TE and TM modes in whispering gallery mode disk resonators as a function of disk radius. The plot compares the situation inside a cell ($n_{cell} = 1.37$; open symbols and solid lines) and in air ($n = 1$; closed symbols and dashed lines). Symbols represent the result of finite element model simulations of nanodisks. Lines represent result of an analytical model for spherical resonators (using a semi-classical approximation for the Riccati-Bessel radial solutions). Nanodisks with diameters in the range used in the present work are seen to provide over ten-fold higher *Q* factors for TE than TM modes, indicating that lasing will likely occur from TE modes.

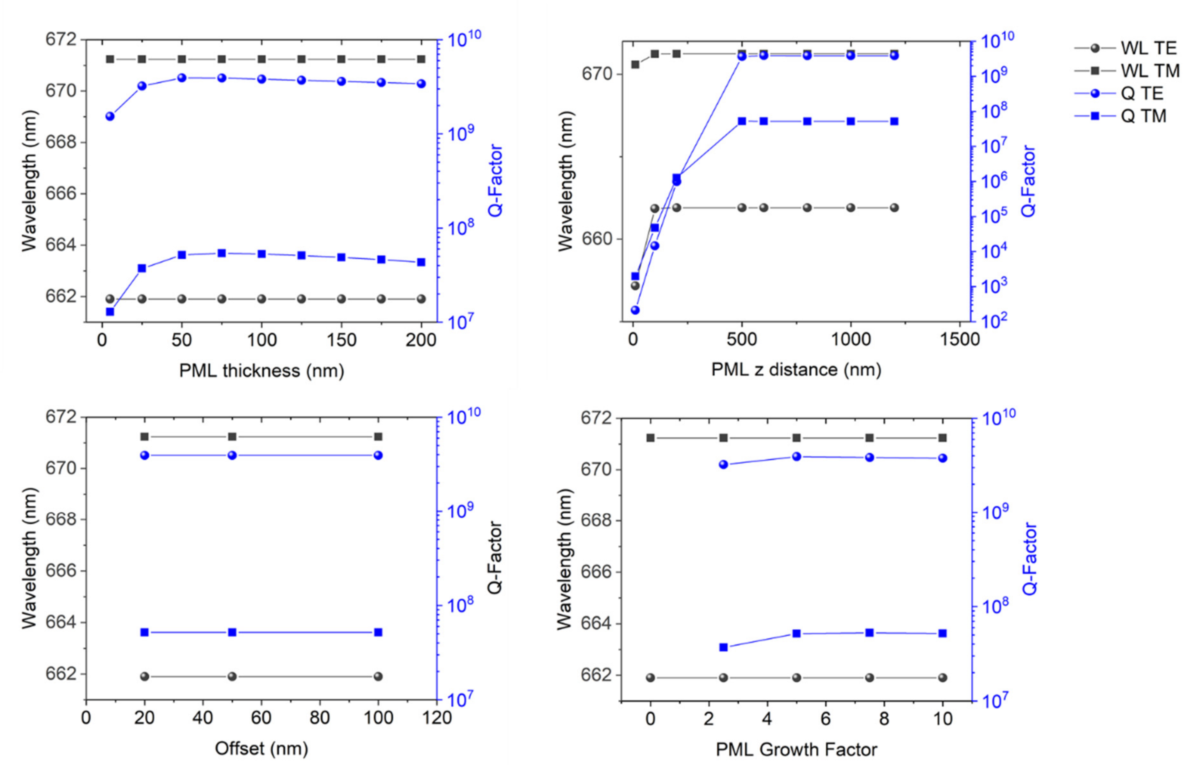

Supplementary Fig. 2. Optimization of perfectly matched layer (PML) conditions to enable extraction of radiative *Q* factors of nanodisks resonators via finite element modelling. PML thickness, z distance, offset and growth factor were varied. The final simulation used conditions where both resonance wavelength and *Q* factor showed weak dependence on PML parameters.

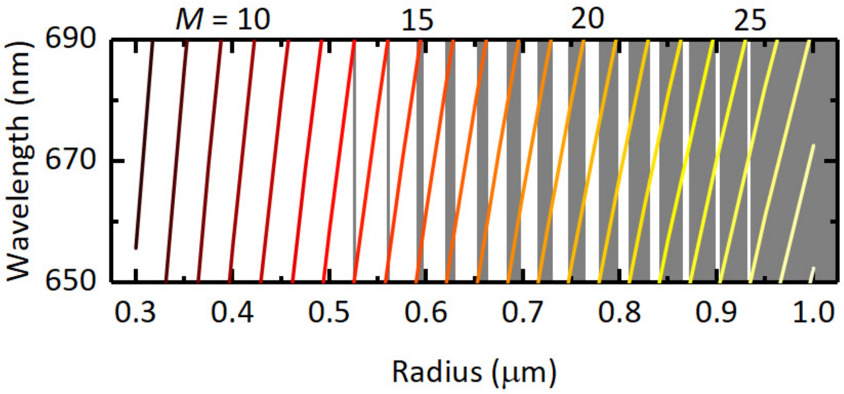

Supplementary Fig. 3. Finite element modelling of the wavelength of the lowest radial order TE modes in whispering gallery mode nanodisks as a function of disk radius. The numbers labelled *M* next to lines indicate the angular quantum number of the corresponding mode. The grey regions indicate disk radii for which multimode emission is expected due to presence of two modes within the gain region of the used material. Disks were assumed to be located in a medium with homogenous refractive index of $n_{\text{cell}} = 1.37$. For a 750 nm diameter disk, an increase in diameter by 1 nm leads to an increase in emission wavelength of 0.84 nm.

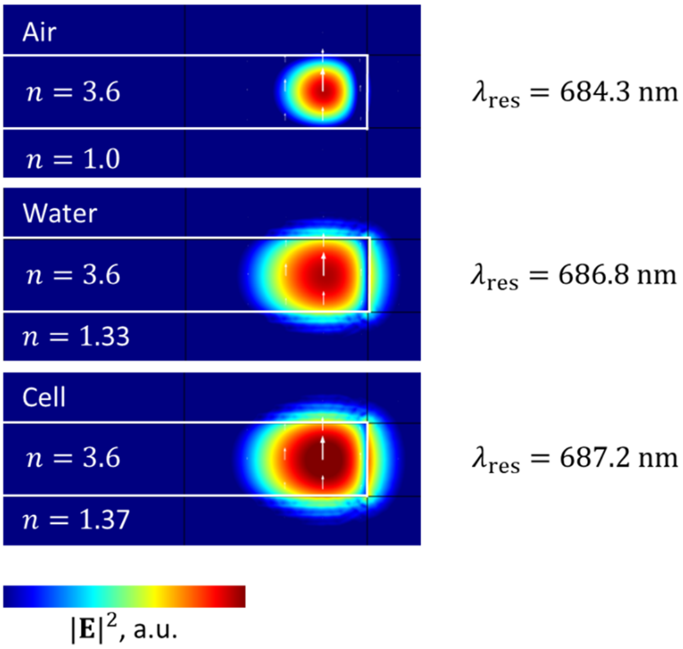

Supplementary Fig. 4. Finite element modelling of a whispering gallery mode nanodisk embedded in environments of different refractive indices (air, water, and inside a cell). False colour maps show $|\mathbf{E}|^2$ (in arbitrary units) of the lowest radial order TE mode that falls within the gain spectrum of the quantum well structure used in this work; λ_{res} indicates the wavelength of this mode in the three conditions. In water and within a cell the resonant wavelength changes by about 10 nm per refractive index unit change of the environment.

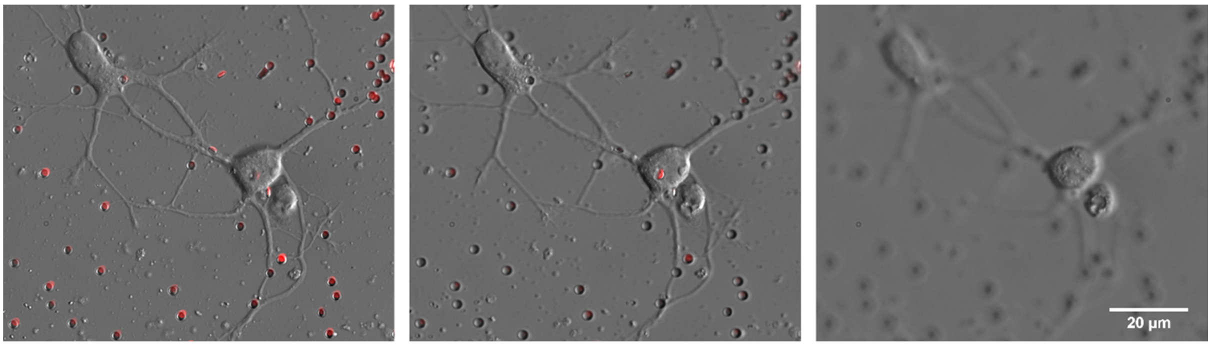

Supplementary Fig. 5. Overlay of DIC microscopy and epi-fluorescence (red) of the primary neuron shown in Fig. 3a of main document. Images were acquired with a 60x oil immersion objective (NA 1.4) and the focus of the microscope was set to three different planes. Left, focussed onto the surface of the culture dish. Centre, focussed at an intermediate plane above the surface and within the soma of two neuron cells. Right, focussed to even higher plane on the top surface of the soma of the right neuron in the field-of-view. Disks surrounding the neurons show bright fluorescence when focusing on the surface of the dish, indicating they rest on the surface. By contrast, the disk seen in the centre of the right neuron is brightest when the focus is set to within the soma. This provides evidence that the disk is located above the surface of the culture dish and thus inside the soma.

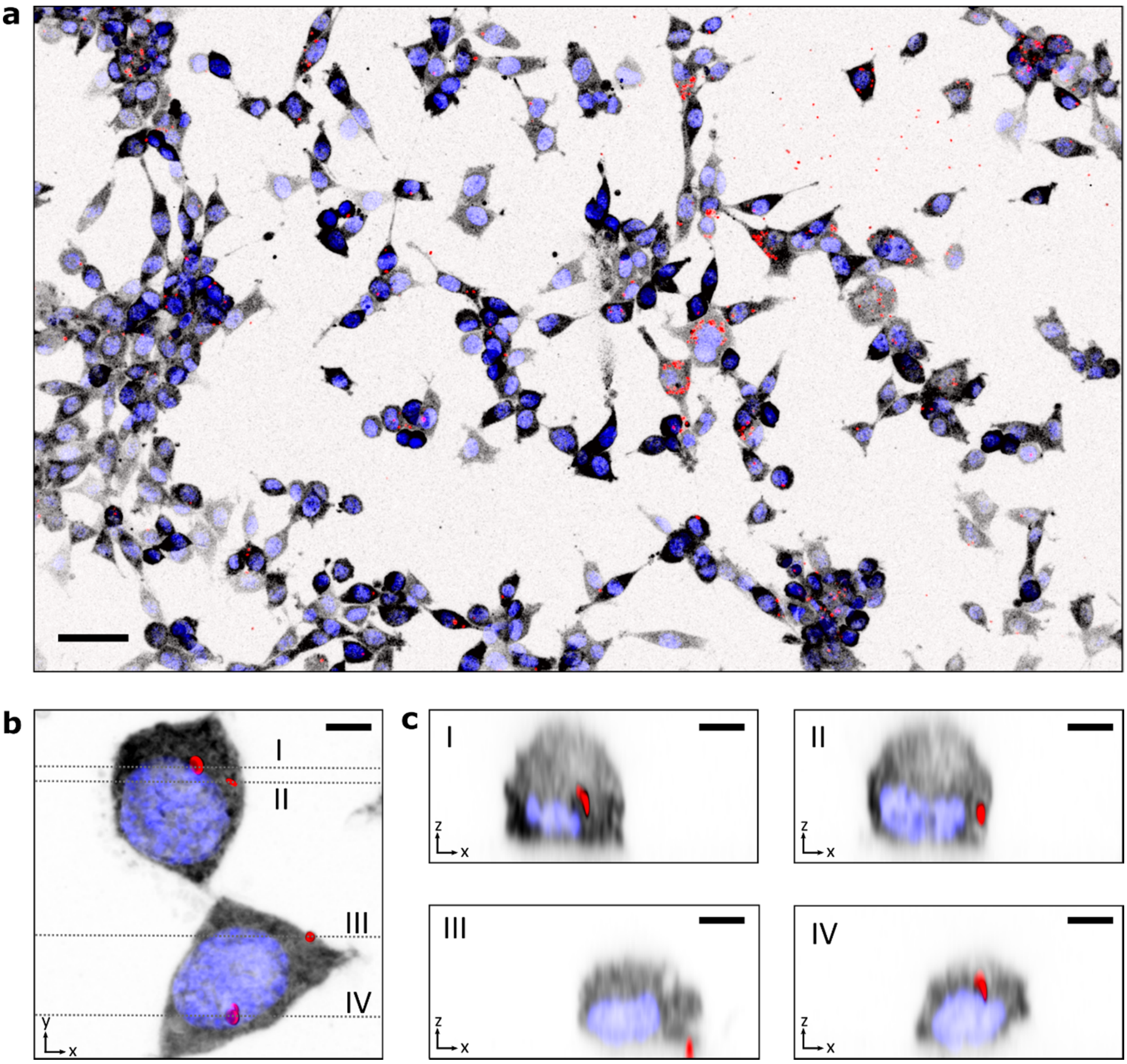

Supplementary Fig. 6. Maximum intensity projection of 3D confocal imaging datasets of nanodisk-labelled NIH 3T3 cells used to confirm internalization of nanodisks and to estimate uptake efficiency. The nucleus and cytosol of the cells are displayed in blue and grey, respectively, while the nanolasers are displayed is red. **a**, Large area scan showing that a high number of cells is labelled with disks, while the surface of the cell culture dish is mostly cleared of nanodisks, except for a small area in the top right corner of the image. Scale bar, 50 µm. **b**, High resolution maximum intensity projection of a scan of two NIH 3T3 cells from the same sample. **c**, 4 cross sections along the dotted lines (I-IV) in b, showing the profile in the x-z plane. Internalized disks (I, II, IV) clearly overlap with the cytosol of the cells while one disk is located at the cell surface (III) and has its maximum intensity below the cell body. Scale bars in (b) and (c), 5 µm. A total of four representative fields of view similar to the dataset shown in panel a (each 750x750 µm^2) were used to estimate the uptake efficiency of disks. All objects in the red channel with an area of at least 200×700 nm^2 (equivalent to the projected area of a nanodisk standing on its edge with respect to the surface of the petri dish) were counted as disks. Disks located below the cell body or elsewhere on the dish surface where categorized as extracellular. Disks located at a position above the dish surface generally overlapped with the cytosol of a cell and were counted as intracellular. This yielded an average uptake efficiency for the nanodisks of (76 ± 5)% (sample mean ± standard deviation) for a total of 1219 counted nanodisks.

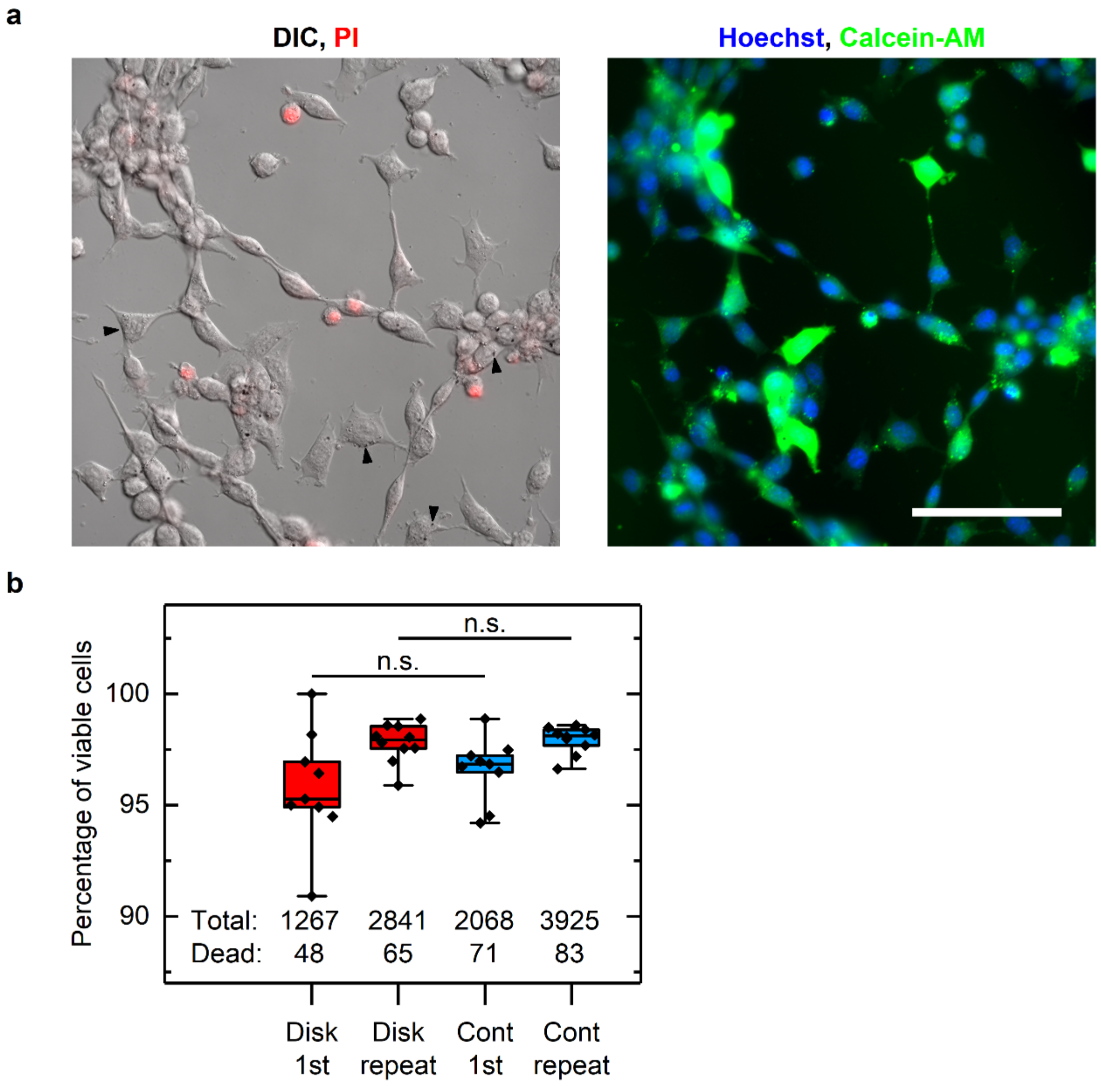

Supplementary Fig. 7. Live/dead assay based on Calcein-AM (live stain) and Propidium Iodide (dead stain) performed on NIH 3T3 cells incubated with and without nanodisk lasers for 96 h. a, Overlay of typical DIC and red Propidium Iodide fluorescence (left) and overlay of green Calcein-AM fluorescence and blue fluorescence from Hoechst labelled nuclei (right). Example shown is for a nanodisk-containing sample; black arrows in DIC/PI image point out representative nanodisks in the sample. Scale bar, 100 µm. **b**, Percentage of viable cells [(Total – Dead)/Total] for two independent experiments (labelled 1st and repeat), each comparing disk-containing cells (Disk, red boxes) to a control without disks (Cont, blue boxes). Each data point represents the percentage of viable cells in a single field of view. Central black line, box and whiskers represent median, 25/75% percentile and 5/95% percentile, respectively. n.s., non-significant difference of populations in t-test.

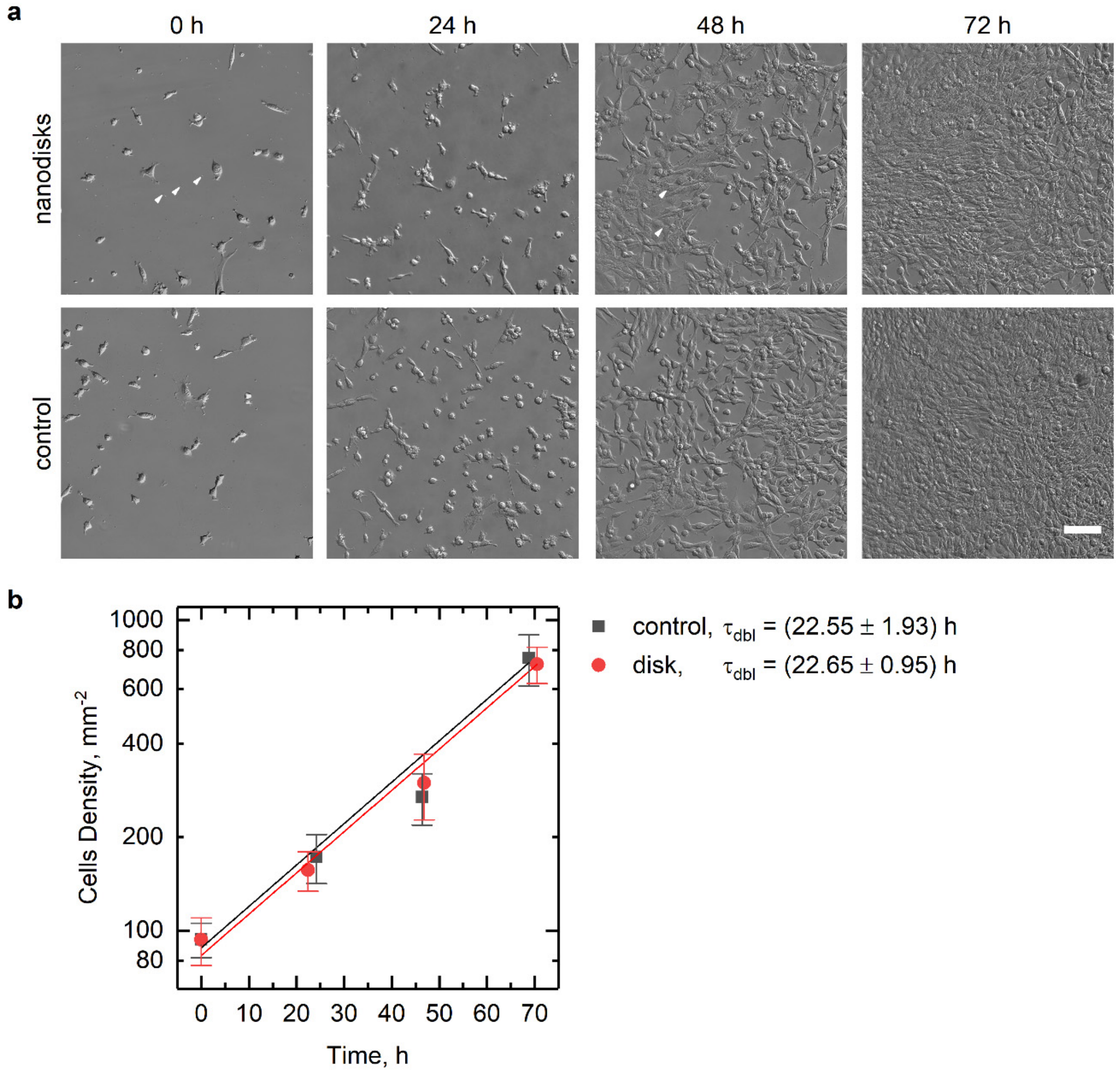

Supplementary Fig. 8. Proliferation experiment on NIH 3T3 cells with and without nanodisk lasers present. a, Typical DIC images for a nanodisk-containing sample and a control sample, imaged at four different time points. White arrows in the nanodisk images point out representative nanodisks in the sample. At 48h, most disks are found to co-localize with cells, indicating internalization (c.f., Supplementary Fig. 6). Scale bar, 100 µm. **b**, Cell density (on log-scale) versus time, as determined by counting cells in nine 670 × 670 µm^2 fields of view for each time point and for each condition (i.e., disk and control). Error bars indicate standard error of the mean (SEM) between different fields of view. Lines are exponential fits [$c(t) = A\ 10^{\beta t}$] to data. The doubling time of cells τ_{dbl} is calculated for each condition from the slope of the fit as $\tau_{dbl} = \log(2)/\beta$. Within the uncertainty of the fit, no significant difference in doubling time is observed between the disk-containing and the control sample. [An independent repeat experiment, performed over a 50 h time course, showed doubling times of (21.4±1.3) h and (24.2±3.6) h for the disk-containing sample and the control sample, respectively.]

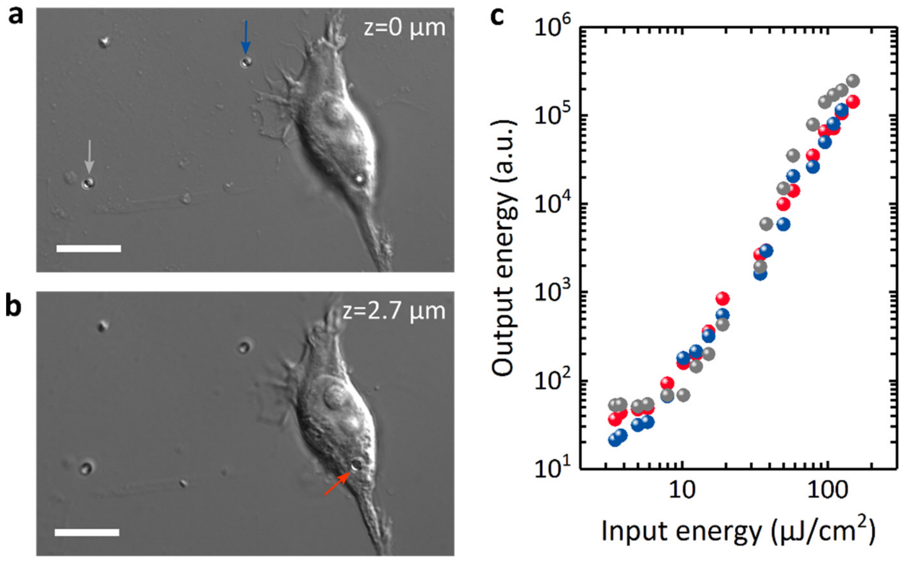

Supplementary Fig. 9. Investigation of lasing threshold for nanodisks located in cell culture medium and located within a fixed NIH 3T3 cell. **a**, DIC microscopy of NIH 3T3 cell sample that was incubated with nanodisk and subsequently fixed. Focus adjusted to surface of culture dish, with two nanodisks resting on the dish surface indicated by blue and grey arrows. **b**, Same field of view as in panel a but with focus 2.7 µm above the culture dish surface, putting an intracellular disk (red arrow) in focus. **c**, Log-log plot of light intensity emitted by nanodisk lasers shown in panels a and b as a function of pump intensity. Colour of symbols correspond to colour of arrows in panels a and b. Scale bars, 10 µm.
