# Supplementary material for: Non-obstructive intracellular nanolasers
Source: Nat Commun. 2018 Nov 16;9:4817. doi: 10.1038/s41467-018-07248-0 (PMC6240115; doi:10.1038/s41467-018-07248-0)
Supplement: Supplementary file 3 — Description of Additional Supplementary Files [file 41467_2018_7248_MOESM3_ESM.pdf]

Description of Additional Supplementary Files

File Name: Supplementary Movie 1
Description: DIC microscopy time-lapse movie of human macrophages incubated with nanodisk lasers. The small dark object in the centre of the field of view is an individual nanodisk. A highly motile macrophage enters the field of view from the right hand side and migrates towards the centre. Upon contact with the nanodisk laser, it internalizes the laser and transports the laser away to the lower left corner of the field of view. Numbers in top right indicate time elapsed after start of time-lapse acquisition in HH:MM.
