# Supplementary material for: Non-obstructive intracellular nanolasers
Source: Nat Commun. 2018 Nov 16;9:4817. doi: 10.1038/s41467-018-07248-0 (PMC6240115; doi:10.1038/s41467-018-07248-0)
Supplement: Supplementary file 6 — Lasing Reporting Summary [file 41467_2018_7248_MOESM6_ESM.pdf]

Corresponding Author Name: Gather

Manuscript Number: NCOMMS-18-21054-T

Reporting checklist for manuscripts with a claim of lasing

In order to promote clarity in claims of lasing we strongly recommend that authors provide the following information when making a claim of lasing action in a new material or device design. We appreciate that in certain circumstances, e.g. random lasers and lasing from nanoscale areas, capturing all recommended data may not be feasible, but we encourage authors to provide the strongest evidence possible. **Please supply a response to the checklist alongside your submitted manuscript, and ensure that the relevant responses are also provided in the main manuscript, methods section or supplementary information as appropriate.** The completed checklist will be shared with reviewers.

Laser data:	Response
1. Have plots of device output power versus pump power been provided over a wide range of values, and do they show a clear threshold?	Yes.
2. Have plots of spectral power density for the emission been provided at pump powers below, around and above threshold and do they indicate clear linewidth narrowing of the emission at the lasing transition? Please state the resolution of the spectrometer used to make the measurements.	Yes.<br>Spectral resolution of spectrometer used was ≈0.2 nm FWHM as declared in main text of manuscript.
3. Has the coherence or polarization of the laser emission been measured?	No. Given the geometry of the lasers and the optical setup this question is not applicable to our system
4. Has the spatial profile of the emission been measured or imaged, and does it indicate the existence of a well-defined beam above threshold?	N/A. WGM lasers do not emit a well-defined beam; this is widely accepted in the literature.
5. Please ensure the pumping conditions (continuous-wave, pulsed, temperature of operation) are clearly stated in the manuscript. In particular, please ensure that threshold values are provided as a density value e.g. W cm^{-2} or J cm^{-2} taking into the account the area of the device.	Done.

(Continues on following page)

Laser data: | Response

6. Have alternative explanations, e.g. amplified spontaneous emission, directional scattering, modification of fluorescence spectrum by cavity, been ruled out as being responsible for the emission characteristics?

Yes.

7. Has theoretical analysis been performed to ensure that the experimental values measured for characteristics such as laser threshold, linewidth, cavity gain–loss and laser efficiency are realistic and reasonable?

To some degree. Lasing has also been observed for similar WGM disk lasers in the past; the novelty of the present manuscript is the integration of these lasers into cells, the presence of lasing is undisputed.

8. How many devices have been fabricated and tested? Can you provide statistics for their behaviour (e.g. time before failure), and estimate any errors that may be present?

During each fabrication run, we produce 5 to 10 million lasers and we have produced about 30 batches of lasers throughout this study. Not all of these lasers have been tested but we provide some statistics, e.g. in Fig. 2b, c, d and Fig. 3b, Fig. 4 as well as via discussion in the manuscript.

Further reading:

We also suggest that authors read the following literature, which describes the important principles and signatures of laser emission, and also discusses some of the common mistakes that can occur during laser characterization.

1. Samuel, I. D. W., Namdas, E. B. & Turnbull, G. A. How to recognize lasing. *Nat. Photon.* **3,** 546–549 (2009).
2. Siegman, A. E. *Lasers* (University Science Books, 1990).
3. Svelto, O. *Principles of Lasers* 5th edn (Springer, 2010).
4. Blood, P. *Quantum Confined Laser Devices: Optical Gain and Recombination in Semiconductors* (Oxford Univ. Press, 2015).
5. Kozlov, V. G., Bulović, V., Burrows, P. E. & Forrest, S. R. Laser action in organic semiconductor waveguide and double-heterostructure devices. *Nature* **389,** 362–364 (1997).
